# Wearable Health Devices in Health Care: Narrative Systematic Review

**DOI:** 10.2196/18907

**Published:** 2020-11-09

**Authors:** Lin Lu, Jiayao Zhang, Yi Xie, Fei Gao, Song Xu, Xinghuo Wu, Zhewei Ye

**Affiliations:** 1 Department of Orthopaedic Surgery Union Hospital, Tongji Medical College Huazhong University of Science and Technology Wuhan China

**Keywords:** wearable, medical field, public health, health monitoring, chronic disease management, rehabilitation.

## Abstract

**Background:**

With the rise of mobile medicine, the development of new technologies such as smart sensing, and the popularization of personalized health concepts, the field of smart wearable devices has developed rapidly in recent years. Among them, medical wearable devices have become one of the most promising fields. These intelligent devices not only assist people in pursuing a healthier lifestyle but also provide a constant stream of health care data for disease diagnosis and treatment by actively recording physiological parameters and tracking metabolic status. Therefore, wearable medical devices have the potential to become a mainstay of the future mobile medical market.

**Objective:**

Although previous reviews have discussed consumer trends in wearable electronics and the application of wearable technology in recreational and sporting activities, data on broad clinical usefulness are lacking. We aimed to review the current application of wearable devices in health care while highlighting shortcomings for further research. In addition to daily health and safety monitoring, the focus of our work was mainly on the use of wearable devices in clinical practice.

**Methods:**

We conducted a narrative review of the use of wearable devices in health care settings by searching papers in PubMed, EMBASE, Scopus, and the Cochrane Library published since October 2015. Potentially relevant papers were then compared to determine their relevance and reviewed independently for inclusion.

**Results:**

A total of 82 relevant papers drawn from 960 papers on the subject of wearable devices in health care settings were qualitatively analyzed, and the information was synthesized. Our review shows that the wearable medical devices developed so far have been designed for use on all parts of the human body, including the head, limbs, and torso. These devices can be classified into 4 application areas: (1) health and safety monitoring, (2) chronic disease management, (3) disease diagnosis and treatment, and (4) rehabilitation. However, the wearable medical device industry currently faces several important limitations that prevent further use of wearable technology in medical practice, such as difficulties in achieving user-friendly solutions, security and privacy concerns, the lack of industry standards, and various technical bottlenecks.

**Conclusions:**

We predict that with the development of science and technology and the popularization of personalized health concepts, wearable devices will play a greater role in the field of health care and become better integrated into people’s daily lives. However, more research is needed to explore further applications of wearable devices in the medical field. We hope that this review can provide a useful reference for the development of wearable medical devices.

## Introduction

### Background

In the 1960s, the concept of wearable technology was first proposed by Edward O Thorp [1], a mathematics professor at the Massachusetts Institute of Technology in the United States. Since then, wearable technology has received considerable attention from researchers all around the world. In recent years, with the development of the internet, intelligent hardware, and big data, wearable technology has developed rapidly in various fields such as health care [2], education and culture [3], social networking [4], and the military [5]. Some of these technologies are becoming part of people’s daily life in the form of accessories such as smart watches, smart bracelets, armbands, and glasses [6]. In the field of health care, wearable devices in the form of portable medical or health electronic devices that can be directly worn on the body can be used to perceive, record, analyze, regulate, and intervene to maintain health and can even be used to treat diseases with the support of various technologies for identification, sensing, connection, cloud services, and storage [7]. By intelligently integrating mechanical functions with microelectronics and computing power, wearable devices can be used to achieve immediate detection of patient signs and laboratory indicators and provide exercise guidance, drug administration reminders, and so on, with the aim of achieving multiparameter, real-time, online, accurate and intelligent detection and analysis of human physiological and pathological information that can be used to carry out self-diagnosis and self-monitoring [8].

As a standard health care intervention, there are 5 main features of wearable devices [9]: (1) wireless mobility; (2) interactivity and intelligence; (3) sustainability and durability; (4) simple operation and miniaturization; and (5) wearability and portability. From the perspective of modern medicine, the application of wearable devices in the field of medicine follows the 4P medical model characterized by preventive, predictive, personalized, and participatory medicine [10]. On one hand, wearable technologies will play a significant role in advancing precision medicine by enabling measurement of clinically relevant parameters showing the health status of individuals [11]. On the other hand, Loncar-Turukalo et al [12] also indicated that wearable medical devices play an important role as an enabling technology and as a key driver that facilitated the emergence of connected health care. The operation and implementation of these devices depend on the application of various wearable technologies, including sensor technology, medical chip technology, wireless communication technology, power management technology, display technology, and information feedback technology [13]. Real-time medical data from these devices are transmitted to the internet for further analyses or feedback from a health care provider.

### Objectives

The development of wearable sensors in the health care market has been relatively slow, despite wearable devices having emerged as a major part of lifestyle and fitness markets. However, the advancement of wearable sensor technology provides enormous opportunities for deployment in health care, especially in connected health care and precision medicine, in which wearable devices can achieve high-quality, real-time measurement of personal health. Although previous reviews have discussed consumer trends in wearable electronics and the application of wearable technology in recreational and sporting activities, data on broad clinical usefulness are lacking. This study reviews the current application of wearable devices in health care. In addition to daily health and safety monitoring, the focus of our work is mainly on the use of wearable devices in clinical practice. We also emphasize their current shortcomings and suggest directions for further research.

## Methods

### Design

A systematic review design with narrative methods was used to analyze the existing evidence. Specifically, a review methodology [14] was carried out to clarify the types of wearable devices and the current status of their use in health care settings.

### Search Strategy

We conducted a comprehensive literature search on January 2, 2020. The following electronic databases were searched with the assistance of an information specialist at the medical library: PubMed, EMBASE, Scopus, and the Cochrane Library. The review was limited to texts published in English between 2015 and 2019 for which abstracts were available. These publication years were chosen due to a dramatic improvement in information technology during that period. The review was also limited to studies of wearable devices in the health care domain. The initial search terms were the following: *wearable devices AND health care*, *wearable technology AND health care*, *sensor AND wearable AND health care*, and *wearable AND track AND health care*. After reviewing the literature identified through these search terms, we added the search terms *monitoring*, *diseases management*, *diagnosis OR treatment,* and *rehabilitation* to capture relevant studies found in the references of the papers retrieved from the initial search.

### Inclusion and Exclusion Criteria

A total of 960 search results were screened for relevance using titles and abstracts, and 82 papers were fully reviewed and are discussed in this study.

Inclusion research criteria were (1) trials including randomized clinical trials and quasi-experimental studies that have proven the effectiveness of wearable devices; (2) studies focusing on clinical applicability; (3) studies published in peer-reviewed journal in English; (4) studies describing completed research; and (5) studies described in full-text papers. There was no restriction on the location of the studies; therefore, international studies written in English were eligible. Exclusion criteria were papers describing the process of the wearable device design, theoretical papers, books or book chapters, letters, statistical reviews, perspectives, dissertations, editorials, and study protocols.

### Study Selection

The research screening process consisted of 4 steps. First, 3 authors (LL, ZJY, and XY) independently screened all titles and abstracts of relevance for this systematic review. Second, the abstracts of all relevant papers were screened for eligibility by the 3 authors. Third, the full texts of eligible publications were obtained and screened (LL) according to the inclusion and exclusion criteria. Differences in opinion were resolved by discussing until a consensus was reached. Finally, to avoid incomplete searching, the references of recent related reviews and the primary studies were manually screened for eligible studies.

## Results

**Figure 1:**
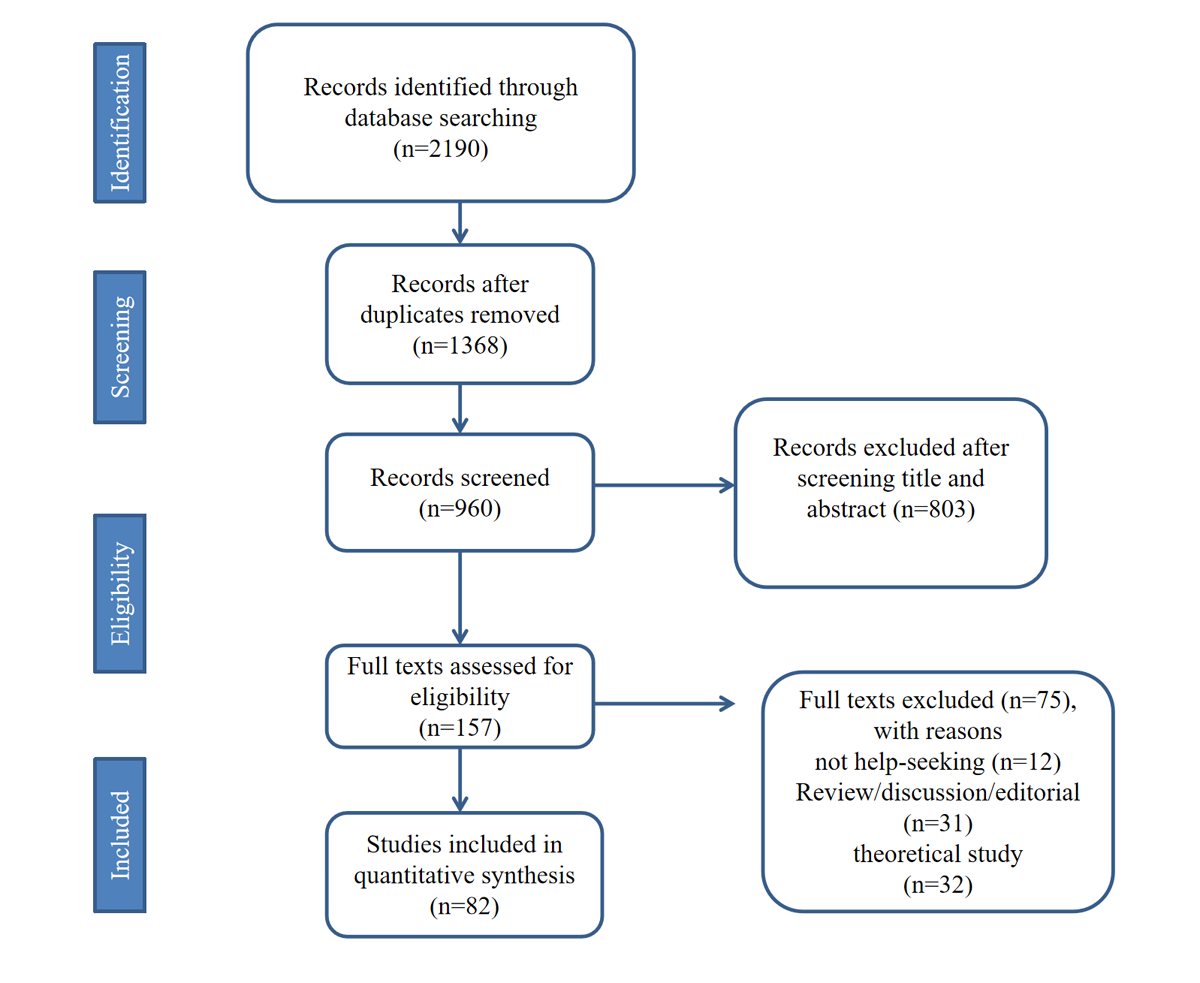
The flow diagram illustrating the screening process of papers.

After reviewing the title and abstract, the search identified 960 potentially relevant documents. Among them, 82 papers met the inclusion criteria after full-text review (Figure 1).

### Classification of Wearable Devices

Wearable devices developed so far have been designed for use on all parts of the human body and are classified into 3 categories: head, limb, and torso wearable devices [15].

Head wearable devices mainly include glasses, helmets, headbands, hearing aids, earrings, earphones, and patches [12]. Among them, Google Glass is a representative type of smart glasses, which has functions such as taking photos, video calls, and GPS positioning, while the application of virtual reality (VR), augmented reality, and mixed reality technology make it suitable for use in areas such as telemedicine, medical education, and intraoperative navigation [16].

Limb wearable devices mainly include wearable devices worn on the arms, legs, and feet [17,18]. Most of the devices worn on the upper limbs are smart watches, bracelets, and other accessories that can be used to monitor physiological parameters such as body temperature, heart rate, ultraviolet exposure levels, and daily activities [19]. Most of the devices worn on the lower limbs appear in the form of shoes and socks that monitor movement-related parameters and are mainly used in the field of rehabilitation [20].

Torso wearable devices mainly include suits, belts, and underwear [21,22]. In recent years, the rapid development of material technology and sensing technology has facilitated the manufacture of electronic products embedded in fabrics or other fabrics that can be used in various biomedical applications. In 2009, a system of clothing providing access to the internet was developed by the Media Technology Laboratory of the Massachusetts Institute of Technology and marked the arrival of a new era of electronic textiles [23].

### Application of Wearable Devices in the Medical Field

In the medical field, wearable devices can connect doctors, patients, clouds, and other parties to understand changes in conditions, to alleviate pain, treat diseases, and facilitate the collection of a large sample of case data, which is helpful for the development of national epidemiology strategies and preventive medicine [24]. These devices are used mainly in health and safety monitoring [25], chronic disease management [26], disease diagnosis and treatment [27], and rehabilitation [28].

### Health and Safety Monitoring

The health and safety monitoring function of wearable devices is mainly used for older adults, children, pregnant women, and patient groups. The wearer’s gait, walking speed, posture, respiratory rate, blood oxygen, heart rate, blood pressure, energy expenditure, position, and other related parameters are monitored in real time to inform nursing requirements [29]. For older adults, a high-quality independent life requires solutions to complex nursing needs related to mobility, intelligence, and independent living, which can be provided by the characteristics of wearable technology [30]. Godfrey et al [31] used wearable devices for gait and fall quantification in older adults, monitoring the viability of older adults’ daily activities in an unattended home environment and recognizing the main types of movement (walking, standing, sitting, lying) to help older adults to live independently. Jung et al [32] developed a wearable fall detection system to detect falls by rapidly uploading data for the position of older adult individuals to the medical center and ensuring timely help and treatment. For children, in addition to detecting routine vital signs for health management, wearable technology is a useful tool for tracking children's daily activities [33]. With the rise of wearable devices, children’s smart watches, bracelets, and backpacks with tracking and positioning functions have emerged in the market. These devices are based on GPS positioning technology to monitor the child’s location and amount of exercise in real time [34]. Furthermore, wearable technology can also be used to monitor mood. Sequeira et al [35] have demonstrated the feasibility of wearable tools in the prediction of depressive symptoms in children and adolescents. Health monitoring of pregnant women can be divided into 2 aspects [36]. On one hand, these applications can be used to monitor the physiological status, emotional state, sleep, and other data before and after conception, while on the other hand, devices can be used to provide immediate feedback on specific problems that occur in the process of child-rearing. Head-med’s Compass Pregnancy Monitor [37] is the first medical-grade fetal health home monitoring product that is easy to operate and monitors maternal heart rate, fetal heart rate, and uterine activity through a disposable abdominal patch. For patient groups, wearable devices can be used to monitor symptom changes during treatment, which can be used for disease monitoring and efficacy evaluations and can contribute to individualization of the treatment plan [29]. For example, wearable sensors can be used to monitor the symptoms of patients with Parkinson disease during drug treatment to help doctors adjust drug doses and evaluate the efficacy of new drugs [38]. van Andel et al [39] used photoplethysmography in heart rate monitoring of patients with epilepsy and showed excellent seizure detection performance. Ryvlin et al [40] developed a wearable device that reliably detects generalized tonic-clonic seizures with high sensitivity and specificity to help physicians optimize antiepileptic treatment and reduce the risk of sudden unexpected death in epilepsy.

### Chronic Disease Management

#### Overview

Chronic disease management involves changing passive disease treatment into active health monitoring [41]. Wearable products facilitate data collection and monitoring throughout the user’s entire day, as well as providing dynamic, intelligent, and comprehensive analysis of various indicators to enable medical treatment of chronically ill patients. This technology also facilitates remote monitoring of diseases, adjustment of remote treatment plans, lifestyle management, and other functions through cloud services, which is of great significance in disease control [42].

#### Cardiovascular Disease

Cardiovascular diseases can be easily overlooked, with sudden and potentially lethal consequences that require emergency treatment [43]. They are often accompanied by changes in myocardial electrophysiology at an early stage [44]. Therefore, improving daily monitoring is important for discovering and controlling heart disease. There are 2 kinds of traditional cardiovascular disease monitoring: invasive and noninvasive. For routine monitoring, noninvasive electrocardiograms (ECG) and Doppler echocardiography are the main means of examining cardiac function [45]. The 24-hour ambulatory ECG (Holter monitor), which is a relatively mature wearable medical device currently used in the clinic, allows dynamic monitoring, which conventional ECG does not. However, due to suboptimal wearing comfort and the fact that the conductive gel used with electrodes can lead to chest skin allergies and ulcers, its application in home-based daily monitoring is limited. Ultrasonic heart examination can only be used in a hospital setting [46]. To allow people to manage their own health, researchers at home and abroad have conducted many studies on wearable health monitoring systems, especially for ECG long-term data collection. Tsukada et al [47] developed a sports vest made from nanofibers coated with an electroconductive polymer to place the ECG electrodes in close contact with the human body. The signal allows display of the ECG signal in real time, and the monitoring data are collected through an app, which increases comfort without risking allergies. The data are uploaded to the cloud and analyzed by a professional physician to realize remote monitoring of heart diseases. In December 2018, Apple developed the Apple Watch Series 4, which combined the functions of ECG and a watch for the first time. In addition, the dial was designed to display a bipolar ECG to monitor occult atrial fibrillation. This device shows similar accuracy in monitoring arrhythmia, atrioventricular block, and QRS duration extension to standard 12-lead ECG recordings [48]. Kaspar et al [49] found that the use of a wearable cardioverter-defibrillator protected patients from sudden cardiac arrest when they were treated in nonhospital settings until reimplantation of an implantable cardioverter-defibrillator.

#### Pulmonary Diseases

Acute exacerbations of chronic obstructive pulmonary disease (COPD) and bronchial asthma can lead to impaired lung function, decreased quality of life, and increased mortality [50]. Active monitoring of the early signs of a patient’s condition and early treatment can prevent these outcomes. The telehealth program aims to facilitate early identification and timely self-management of acute exacerbations of COPD and bronchial asthma [51]. For such patients, early detection of progression can help to control the disease [52]. The emergence of inexpensive wearable devices has enabled people to monitor heart rate, pulse, oxygen saturation, and physical activity continuously, as well as audio to detect cough, breath sounds, and other characteristics [53]. These signals can be used in predictive analyses to detect early deterioration of lung function. A prospective cohort study [54] conducted at the University of Toronto evaluated a wearable system that reliably captured nearly continuous patient respiratory rate, oxygen saturation, heart rate, and other data for screening of early COPD deterioration. The results of this study demonstrated the feasibility of using a smart watch for centralized monitoring of patients with COPD. A wearable device developed by Colantonio et al [55] uses a wireless sensor network system to monitor the patient’s respiratory rate, respiratory sound, blood oxygen saturation, and ECG to evaluate the therapeutic effect of treatments for COPD. Li et al [56] used acoustic respirators to monitor nighttime wheezing in asthmatic children and found that 57% of patients with well-controlled asthma had a significant number of nighttime wheezing episodes and poor association with routine measurements of lung function. This has helped to develop individualized treatment for children with asthma. However, Rubio et al [51] reported disappointing results for the use of wearable devices to monitor acute exacerbations of COPD, in part because the parameters monitored (symptoms, pulse oximetry, and lung volume) were not reliable indicators for predicting exacerbation of the disease. It should be pointed out that with advances in sensing technology, wearable systems can also link individual environmental exposure with physiology and subsequent adverse health reactions, providing clues for the pathogenesis of certain lung diseases [57].

#### Diabetes

Diabetes represents a group of metabolic diseases characterized by hyperglycemia caused by defects in insulin secretion or impaired biological effects [58]. Long-term poor glycemic control can lead to damage, dysfunction, and failure of various organ tissues, especially the eyes, kidneys, nerves, heart, and blood vessels [59]. For patients with diabetes, improving the ability of self-monitoring and self-management of blood glucose levels has contributed to the reduction of diabetes-related morbidity and mortality. There are currently 3 types of medical management products on the market for patients with diabetes: blood glucose level monitoring equipment, injectable insulin, and implantable insulin pumps [60]. Among them, blood glucose level monitoring products have an important position in blood glucose level control, which is the basic reference for the adjustment of other treatment methods and can also prevent the occurrence of risk events [61]. Traditional blood glucose level monitoring is performed by directly drawing a venous blood sample or taking a finger-prick blood sample, which is analyzed by a biochemical analyzer. These methods are invasive and inconvenient, especially for patients with diabetes who need to monitor blood glucose level several times a day. Due to the volatility and transience of blood glucose level testing, traditional single-point testing methods do not truly reflect the changes in glucose levels in the body [62]. With the development of mobile and sensor technologies, wearable dynamic blood glucose level monitoring products have emerged. The GlucoWatch [61], a noninvasive, painless blood glucose level monitoring product approved by the US Food and Drug Administration in March 2001, has proven its applicability and feasibility in the field of diabetes monitoring products. At present, wearable medical devices widely use indirect measurement methods (minimally invasive or noninvasive) to measure parameters such as blood glucose concentration. The main methods are spectrometry, blood substitution (urine, tears, and tissue fluid), counter-ion electroosmosis, and microwave technology [63,64]. Compared with other methods, the optical method is rapid, is noninvasive, is nonpolluting, is simple to operate, and has become the main method for noninvasive blood glucose level detection. The principle of the measurement is based on concentration-dependent changes in the absorption and reflection characteristics of glucose [65]. However, the accuracy of the measurement is limited by the overlap of other blood components absorption spectra with the absorption spectrum of glucose [64]. In addition, Medtronic’s Minimed 670G uses a portable design to integrate blood glucose level monitoring and an insulin pump. The patient can affix the device to the waist and set the daily detection and injection times. Pillalamarri et al [66] developed a handheld insulin pump using biomedical microelectromechanical systems technology to intelligently control the rate and volume of insulin injections, maintaining blood glucose level within a relatively stable range.

#### Hypertension

Hypertension is a chronic disease characterized by a sustained increase in arterial blood pressure. It is the most important risk factor for cardiovascular and cerebrovascular diseases, which seriously endanger human health [67]. Therefore, the accuracy and reliability of blood pressure measurement during the diagnosis and treatment of hypertension is especially important [68]. Blood pressure can be measured directly and indirectly [69]. Direct measurement involves percutaneous puncture and catheterization of the aorta. This is an invasive method and is only applicable in critical and difficult cases. Indirect measurement, also known as cuff compression, involves the use of a sphygmomanometer. This is the most commonly used measurement method, but continuous data cannot be obtained [70]. Noninvasive continuous blood pressure measurement is the trend for the development of wearable blood pressure monitors. At present, wearable devices determine blood pressure by measuring different physiological signals and can be divided into 4 types according to the principle [71]: (1) flattening tension of the radial artery; (2) volume changes in pulsing blood; (3) speed of pulse wave; and (4) the vibration measurement method. Moreover, wearable blood pressure monitor can be roughly divided into 2 types according to structure: cuff type and sleeveless type. Due to its strong anti-interference and reliability, the cuff type has become the mainstream form of wearable blood pressure measuring devices and are widely used in the market [72]. This type of wearable device uploads the monitored data to generate a dynamic blood pressure map, which is convenient for patients and doctors. However, during daily continuous monitoring, repeated inflation and deflation of the cuff can cause physical discomfort to the patient, especially at night when the process can cause sleep disturbances [70]. Therefore, a wearable device that can measure dynamic blood pressure accurately and comfortably without a cuff would be attractive prospect. Zheng et al [73] used a wearable sleeveless device developed based on optical technology to monitor blood pressure changes by measuring the pulse arrival time (the pulse transit time from the heart to the peripheral blood vessels). In a mixed methods study, Islam et al [74] demonstrated that wearable blood pressure device measurements compared well against a gold-standard ambulatory device, indicating that this user-friendly method has the potential to enhance blood pressure management in long-term monitoring. However, this type of sleeveless equipment is still in the experimental development stage and has not yet entered the market.

### Diagnosis and Treatment of Diseases

#### Overview

A comprehensive understanding of the changes in physiological and pathological indicators during the early stage of disease is critical for timely diagnoses and interventions. Wearable devices are of great significance in the diagnosis and treatment of various diseases by monitoring the changes in vital signs on a real-time basis [42].

#### Neurological Disorders

For example, early warning and intervention in the predementia phase of Alzheimer disease are of great significance in delaying the onset and reducing the incidence [75]. Mild cognitive dysfunction is a major feature of predementia in Alzheimer disease, and diagnostic methods at this stage are not yet fully developed [76]. Recent studies have shown that gait is a noninvasive biological indicator of cognitive function [75,77,78]. By wearing a wearable device, the user’s gait parameters can be collected for early detection of Alzheimer disease. In addition, wearable devices also show good application prospects in the early diagnosis of other neurological diseases. For instance, Mannini et al [79] developed a wearable device that analyzes gait classification to improve the accuracy of diagnosis of early neurological diseases.

#### Respiratory Diseases

For patients with obstructive sleep apnea hypopnea syndrome, the application of wearable nocturnal breathing monitoring equipment can improve the accuracy of early diagnosis and can be used at home [80]. Surrel et al [81] developed a wearable, accurate, and energy efficient system for monitoring obstructive sleep apnea on a long-term basis.

#### Cardiac Conduction System Anomalies

For patients who may be at risk of cardiac arrest, a wearable defibrillator can help monitor arrhythmias. Moreover, emergency defibrillation can be performed to restore normal rhythm when cardiac arrest or ventricular fibrillation is detected [82]. The annual meeting of the American Association for Cancer Research in 2017 also reported that a medical wearable device that delivers alternating currents extends the overall survival of patients with malignant gliomas [83].

#### Urinary Diseases

For end-stage renal disease, Gura et al [84] showed that treatment with a wearable artificial kidney was well tolerated and resulted in effective uremic solute clearance and maintenance of electrolyte and fluid homeostasis.

However, wearable devices still have a long way to go in terms of therapeutic applications compared to their health monitoring functions. It should be pointed out that in recent years, with the rise of VR, augmented reality, and mixed reality technology and the breakthrough of remote technology, wearable equipment has also undergone significant developments for application in settings such as medical education, the formulation of preoperative surgery plans, intraoperative navigation, preoperative doctor-patient communication, and remote consultation [16]. In our previous studies [16,85-87], we successfully implemented mixed reality technology to facilitate preoperative communications between doctors and patients, intraoperative guidance, remote surgical consultation, and surgical navigation through HoloLens glasses.

### Rehabilitation

#### Overview

In the field of rehabilitation, wearable devices are used mainly in sports rehabilitation [88], cognitive rehabilitation [89], and as rehabilitation aids for people with disabilities [90].

#### Sports Rehabilitation

Conditions such as stroke, brain trauma, spinal cord injury, and musculoskeletal injury often lead to the loss or decrease of a patient’s motor ability. The main goal of sports rehabilitation is to restore balance and coordination, to ensure normal joint mobility, and to have sufficient muscle strength and muscular endurance for daily life [91]. Traditional sports rehabilitation is mainly performed in specific medical institutions by professional rehabilitation practitioners who carry out training to expand the range of motion of joints, enhance muscle strength and endurance, and improve balance and coordination function. This mode of rehabilitation training has the advantages of safe reliable methods and real-time guidance from professionals [92]. At the same time, there are some shortcomings in the traditional rehabilitation model that cannot be ignored, such as limitations in the time and place of rehabilitation, as well as the boredom and tedium of the process, resulting in lack of adherence among patients, even those who are not older adults, all of which seriously affect the efficacy of sports rehabilitation [21]. The application of wearable devices, especially in combination with VR, augmented reality, and mixed reality technologies, not only offers the ability to comprehensively monitor and evaluate the rehabilitation activities of patients but also to make the activities more interesting and improve patient adherence [92].

Limb hemiplegia after brain injury is a difficult problem in sports rehabilitation [93]. According to The Chinese Stroke Prevention Report in 2018, stroke has become the leading cause of death in China and the leading cause of disability in Chinese adults [94]. In an aging society, the incidence of stroke is expected to increase in the coming years. Among the various defects caused by stroke, unilateral sensorimotor deficits are very prominent, and 80% of stroke patients have different degrees of gait abnormalities [95]. At present, lower limb rehabilitation for stroke patients is focused mainly on gait training [28]. The wearable device can be used to monitor the patient’s gait parameters and provide feedback to help the doctor assess the patient’s recovery in real time so that the treatment plan can be adjusted accordingly. Hsu et al [96] used a multiplaced wearable sensor to analyze and classify gait characteristics in patients with neurological disorders to guide the selection of rehabilitation exercise regimens. Furthermore, in cases of hemiplegia of a single limb caused by neurological disease, the recovery of arm function lags behind other functions, although the posture and gait may be significantly improved [97]. Maceira-Elvira et al [98] used a wearable stroke rehabilitation trainer to support patients performing a personalized upper limb neuromotor training program at home. The built-in wireless sensor records the patient’s exercise volume, analyzes the data and feeds the data back to the patient and therapist, thus bringing additional improvement to the recovery of the patient’s upper limb functions. Using an optimized wearable glove, a team from the University of Pisa used hand posture reconstruction technology to reconstruct hand movements, allowing real-time recording and feedback on a patient’s hand function recovery [99].

Moreover, in a broad sense, the rehabilitation treatment of spinal cord injury and musculoskeletal injury can be categorized as orthopedic rehabilitation. The use of orthopedic aids is particularly important in the treatment of modern orthopedic rehabilitation. For patients with spinal cord injury, early rehabilitation treatment leads to better the recovery of spinal nerve function mainly through improvements in nerve plasticity [100]. Traditional rehabilitation treatment, which is guided mainly by professional rehabilitators in hospitals, is a costly process requiring a long period of hospitalization. Furthermore, the qualification requirements of the rehabilitation professionals are often high, and one-on-one professional guidance is not always possible. Many researchers [101,102] have designed wearable artificial exoskeletons based on bionics, the principle of which is to transfer the human body load to the artificial exoskeleton and assist in maintaining the standing posture of patients with spinal cord injury. This approach is also designed to allow the patient to walk slowly with a fixed gait maintained by the dynamics and feedback systems of the artificial exoskeleton, thus avoiding joint dysfunction such as joint stiffness and muscle contracture [103]. On the other hand, 3 factors are key to the success of treatment of musculoskeletal system injury: reduction, fixation, and functional exercise [104]. The rehabilitation exercises in the later stage after the injury are the most important. Many orthopedic surgeons in clinical practice often consider only the aesthetics of the operation, neglecting the importance of postoperative limb function rehabilitation. Furthermore, many patients pay insufficient attention to postoperative rehabilitation exercise, with poor adherence, and fear of pain, all of which affect the recovery of the function of the affected limb and greatly reduce the effectiveness of the surgery. The use of wearable devices can encourage patients to perform rehabilitation exercises and allow adjustment of the intensity of rehabilitation training to improve training results based on feedback information [103]. The electronic-assisted instrument system developed by Zhu et al [105] promoted the recovery of knee flexion and extension function in patients after total knee arthroplasty. Lee et al [106] designed an exoskeleton suit that can assist multiple joint movements and measure the direction and angle of movement. Through intuitive recording of the data, which is displayed in a graphical form, the exoskeleton suit provides patients and physicians with information about the effectiveness and extent of the exercise, which is conducive to early rehabilitation and restoration of function.

#### Cognitive Rehabilitation

Cognitive dysfunction, which is one of the most common sequelae after brain injury, not only influences the quality of life of patients but also puts tremendous pressure on patients’ families and society [107]. Therefore, improvements in rehabilitation methods are of great significance for cognitive dysfunction. VR glasses have shown great potential in providing treatment options and assessment tools for patients with cognitive impairment [108-110]. VR technology has 3 characteristics, namely immersion, interactivity, and imagination [111]. By providing visual, auditory, and tactile sensory simulation, patients are provided with an immersive experience that aids cognitive rehabilitation in a controlled stimulation environment and facilitates monitoring of related parameters in real time. VR technology offers the potential to develop a personalized treatment plan for patients with different levels of cognitive impairment by providing virtual reproducible images, which are effective in the recovery of memory impairment. To date, many studies have demonstrated the value of VR-based wearable devices in the rehabilitation of cognitive impairment. According to the characteristics of immersion, Faria et al [112] designed VR glasses to perform memory recovery training in patients with cognitive dysfunction, reducing their fear of reality and improving their learning and behavioral abilities. Wåhlin et al [113] used VR training to improve left-sided awareness in chronic stroke patients also increased sporadic interhemispheric functional connectivity within the dorsal attention network.

#### Rehabilitation Aids for People With Disabilities

Accessories for people with disabilities is another major direction in the field of wearable devices for rehabilitation. Such accessories include intelligent glasses for the blind, smart hearing aids, and intelligent prosthetics.

Using artificial intelligence technology, smart glasses for the blind [114] not only help individuals with visual impairments interpret information about their surroundings (identified by a pinhole camera, eg, to analyze road condition information in real time) but also aid in making effective decisions. Moreover, smart glasses can help individuals with visual impairment to use a variety of smart home products in a family setting, thus allowing them to improve their quality of life [115].

The hearing aid is essentially an electroacoustic amplifier [116]. The acoustic signal is converted into an electrical signal by a microphone, and after amplification, the electrical signal is restored by the receiver to an acoustic signal and transmitted to the human ear. The emergence of intelligent technology has promoted the development of the hearing aid industry, allowing users to autonomously choose the clearest sound they want to hear in a complex and changing environment, freeing them from the disadvantages of hearing disorders.

For patients with limb defects, the prosthesis can not only fill the shape defect but also restore full or partial residual limb function [117]. Traditional prosthetics are cumbersome, and the invention of wearable smart prostheses is very encouraging for patients with physical disabilities. Intelligent prostheses incorporating robotics have become a hot topic in recent years [118].

## Discussion

### Existing Issues

#### Overview

The development of wearable medical equipment has increased the popularity and quality of health care. Moreover, these developments have, to a certain extent, alleviated the shortage of medical resources in low-income countries and promoted the development of medical care worldwide. However, the wearable medical device industry is still in development and currently faces several important limitations that prevent further use of wearable technology in medical practice.

#### Barriers to User-Friendly Solutions

For the health care system, the main challenge is to enable the use of these technologies by changing the model of care and by sharing information [119]. Data collection, transfer, preservation, and sharing require not only the development of technical solutions but also the development of legal infrastructure, which will enable different organizations to share data and responsibilities for patient [120]. On one hand, patient autonomy in using wearable health devices needs to be kept to help patients to become active participants in their own care [119]. On the other hand, the duty of clinicians and the responsibilities for misdiagnosis and missed diagnosis due to the use of unreliable or delayed data and false alarms during the use of wearable devices should also be legally regulated [121].

#### Security and Privacy Concerns

Through sensor technology, wearable health devices can collect all kinds of user information, such as health information, geographical location, and living habits [34]. The various formats, large scale, and numerous mobile links of these data may increase the risk of leakage and tampering [8,35,122,123]. Strategies to ensure the security of the data and improve the public trust are required.

#### Lack of Industry Standards and Regulations

In the absence of industry standards and regulations, all companies hope to rely on their core products to form their own standards and regulations, making it difficult to integrate resources. So, the establishment and enforcement of new regulatory standards are required [71,118].

#### Technological Barriers

There are many technical bottlenecks in the applications of wearable medical devices [64,124,125]: (1) Data accuracy [9,24,118,122]: On one hand, the sensor specificity of current wearable device is low, which may lead to overdetection of benign nonclinical related signals, resulting in misdiagnosis, unnecessary examinations, and patient anxiety. On the other hand, low sensor sensitivity may lead to omission of pathological clinically-relevant parameters, resulting in missed diagnosis and delay in treatment. (2) Single function: The compatibility of wearable devices is relatively poor, the functions are mainly concentrated on the level of health monitoring, with slow progress in clinical treatment, and few wearable medical devices have effectively integrated multiple functions [27,124]. (3) Poor battery life: Designing low-power consuming and high-energy storage wearable devices has always been an exciting but challenging issue [10,120]. (4) Equipment safety: Safety and security are primary considerations for medical equipment, closely related to reliability at all system levels [12,13]. Because false alarms would reduce user alertness and prevent user adherence from the feedback provided, efficient mechanisms are needed to detect and diagnose deviations occurring in captured data [126]. (5) Other issues such as cost, low data collection and processing efficiency, unstable human-computer interaction interfaces, and incomplete construction of big data health clouds need to be further improved [9,13,122].

### Limitations

There were some limitations in our review. Although some databases are included, our search terms may cause the omission of relevant papers. Due to the exploratory nature of this review, it included a wide range of research designs, and the review will ultimately be limited by the design of the included studies. Although the second and third reviewers used strategies to limit bias through consultation, they also acknowledge the possibility of subjectivity in analyzing the survey results. Additionally, this paper is not intended to be a systematic review, and it is possible to conduct a broader review and find papers suggesting other applications of wearable devices.

### Conclusion and Future Directions

Despite many limitations in their application, wearable devices have achieved remarkable success and have brought huge benefits to the aging global population. The authors believe that the following aspects are crucial for future development of the wearable device industry:

1. Strengthening oversight of the wearable device industry, formulating specific security rules to protect the privacy and security of personal data, and clarifying the relevant medical responsibilities and rights between doctors and patients.
2. Establishment and enforcement of industry standards by taking health care as the main body of information to establish a unified data classification, evaluation system, and industry standards for wearable device to realize the mutual recognition of medical data.
3. Technological advancement in wearable health devices to develop low-consumption and high-integration sensor technology; low-power high-performance battery technology; high processing efficiency medical chip technology; and human-computer interaction technology to improve information accuracy, information processing speed, extend battery life, and user experience.
4. Combining big data, cloud computing, and IoT—the internet of things—to build a healthy database to develop a complete medical ecosystem. Using this resource, we can fully develop, analyze, and employ medical and health big data to expand the use of wearable devices in other fields, such as telemedicine, preventive medicine, and epidemiology [12].

By combining smart wearable medical devices with pension services, a smart retirement community can be built to provide high-quality, high-efficiency medical care services. How medical services are sought has also begun to transform from passive medical treatment of disease to community medical models led by prevention, health care, and prediagnosis.

In summary, with the development of science and technology and the popularization of personalized health concepts, wearable devices will inevitably play a greater role in the field of health care and become better integrated into our daily lives. However, more research is needed to explore further applications of wearable devices in the medical field.

## Abbreviations

VR: virtual reality
ECG: electrocardiogram
COPD: chronic obstructive pulmonary disease
